# Effect of Milk Origin and Seasonality of Yogurt Acid Whey on Antioxidant Activity before and after In Vitro Gastrointestinal Digestion

**DOI:** 10.3390/antiox12122130

**Published:** 2023-12-18

**Authors:** Eleni Dalaka, Georgios C. Stefos, Ioannis Politis, Georgios Theodorou

**Affiliations:** Laboratory of Animal Breeding and Husbandry, Department of Animal Science, Agricultural University of Athens, 11855 Athens, Greece; elenidalaka@aua.gr (E.D.); i.politis@aua.gr (I.P.)

**Keywords:** strained yogurt, dairy by-product upcycling, antioxidant biochemical assays, cellular assays, HT29, THP-1, bioactive peptides

## Abstract

Yogurt acid whey (YAW) is a by-product of Greek strained yogurt production. The disposal of YAW constitutes an environmental problem, and given the increasing demand of Greek yogurt worldwide, its handling is a challenge. However, whey-derived peptides, resulting from microbial fermentation as well as those resulting from further hydrolysis during the digestion process, have been linked to enhanced biological activities. In this study, the antioxidant capacity of 33 samples of YAW obtained from Greek dairy companies of bovine, ovine or caprine origin was investigated using both cell-free and cell-based assays. The YAW samples, their in vitro digestion products (YAW-Ds) and a fraction of the digests (less than 3 kDa; YAW-D-P3) were assessed using four biochemical assays, namely ORAC, ABTS, FRAP and P-FRAP. Our data revealed a higher antioxidant capacity for digested samples compared with undigested samples, with all four methods. ORAC values after in vitro digestion were higher for the ovine samples compared to their bovine (YAW-D and YAW-D-P3) and caprine (YAW-D-P3) counterparts. Furthermore, the YAW-D-P3 fraction derived from samples collected in the summer months exhibited higher ORAC values when compared to the respective fraction from the winter months’ samples. The cellular antioxidant activity of ovine YAW-D-P3 was improved in H_2_O_2_-treated HT29 cells compared to the control H_2_O_2_-treated cells. However, YAW-D-P3 could not trigger either the pathways involving the transcription factors NF-κB or NFE2L2 or the gene expression of SOD1, CAT and HMOX1 in LPS-challenged THP-1-derived macrophages. These results suggest that YAW, and particularly YAW from ovine origin, could be used as a natural source for its antioxidant potential in human and animal nutrition.

## 1. Introduction

Strained yogurt, commonly referred as Greek-style yogurt, has gained immense popularity due to its taste and high nutritional value [1]. The production of Greek yogurt results in large volumes of yogurt acid whey (YAW; for every 1 kg of Greek yogurt, 2–3 kg of YAW are produced [2]) as a by-product of the process, which cannot be readily utilized nor easily disposed [3]. For instance, due to its high lactic acid content, it is difficult to dry YAW or extract its lactose [4], while it has a high biological and chemical oxygen demand, ranging from 45,800 to 50,500 mg/L and 52,400 to 62,400 mg/L, respectively [5]. Currently, YAW is incorporated into some food products and used for biofuel production, animal feed and land application [1]. However, the dairy industry has been making constant efforts to develop innovative methods for upcycling YAW in a sustainable manner [6,7]. Despite the inherent challenges of using YAW due to its nature, it is an appealing material since it has a high mineral content and lactose as well as small amounts of protein [5,8].

The trend of healthy nutrition and well-being has shifted consumers’ interest towards products that are high in protein and peptides. Although the protein content of YAW is low, the total amount of protein contained in the large volumes of resulting YAW should not be ignored. Furthermore, given the higher solubility and biological value of whey proteins over caseins [9], there is a basis in the idea that YAW’s proteins/peptides are also of high quality. It is crucial to mention that YAW has been suggested as a potential source of bioactive peptides [9,10].

Bioactive peptides of whey are known for their antioxidant activity, although their function is not fully elucidated [11]. Researchers have pointed out that peptides can inhibit lipid oxidation [12], inactivate reactive oxygen species (ROS) and scavenge free radicals [13], as well as chelate metal ions by eliminating traces of metals to facilitate oxidation [14]. Moreover, they can modulate important transcriptional regulatory pathways and stimulate the synthesis of antioxidant cell defense compounds [15]. The antioxidant properties of peptides are related to their amino acid composition, chemical structure and hydrophobicity [16].

For assessing the antioxidant activity of peptides, both cell-based and cell-free methods exist. Concerning the latter, given the differences in the mechanisms of action of the different antioxidant measurement methods, a single antioxidant assay can produce a relative result but is not capable of elucidating the actual antioxidant capacity of a complex sample [17]. Biochemical antioxidant assays can be divided into two major categories: assays based on hydrogen atom transfer (HAT) reactions and assays based on electron transfer to probe molecules (ET) [18]. HAT-based assays measure the capability of an antioxidant to scavenge ROS by hydrogen donation in kinetic time [19]. In contrast, ET mechanisms identify the ability of an antioxidant to provide one electron to the free radical [20]. In our study, we used one HAT assay, oxygen radical antioxidant capacity (ORAC), and three ET assays, 2,2′-Azinobis (3-ethylbenzothiazoline-6-sulfonic acid) radical scavenging assay (ABTS), ferric reducing antioxidant power (FRAP) and potassium ferricyanide reducing power (P-FRAP).

Nowadays, a considerable number of studies have focused on cell-based assays using different cell lines or isolated cell models under diverse oxidative stress, as they are rapid, inexpensive and reliable [21]. The cellular antioxidant activity (CAA) method is more appropriate than traditional cell-free methods, as it exhibits greater biological relevance and reflects the capacity of antioxidants to reduce intracellular oxidative stress, since it takes into account the participation of the different components of the cell that are critical to develop an antioxidant response [22]. Intestinal (Caco-2 and/or HT29) cellular lines have been broadly used for the measurement of CAA [23,24].

A major player in mediating the response to oxidative stress is the transcription factor NFE2L2, which exerts its important role through binding to antioxidant-responsive elements (AREs) to regulate the expression of genes encoding for proteins with cytoprotective roles [25,26]. Among these proteins, the following are included: the markers for cellular antioxidant defense system, catalase (CAT) and superoxide dismutase 1 (SOD1), which [27] represent the indirect antioxidants, as well as HMOX1 (heme oxygenase-1), which represents a prime cellular defense mechanism [28]. The nuclear factor kappa-light-chain-enhancer of activated B cells (NF-κB) pathway has been suggested as an atypical pathway against oxidative stimuli [29]. The NF-κB transcription factor consists of the NF-κB and REL subfamily proteins, with major subunits p50 and p65, respectively. The canonical (also known as classical) signaling pathway is activated by pattern recognition receptors like the toll-like receptors and leads to the activation of NF-κB target genes, p50 in conjunction with RELA [30]. ROS can both activate and repress the NF-κB signaling pathway; thus, it may have both anti- and pro-oxidant roles in modulating oxidative stress.

However, in the literature, there are limited studies that have focused on the antioxidant capacity of dairy by-products, while there is a gap concerning data on the biofunctional properties of YAW. In an overall effort of our group to evaluate YAW as a potential food or feed component [31,32,33], the aim of the present study was to examine the effect of species of milk origin and seasonality on the antioxidant capacity of YAW samples obtained from Greek dairy companies. The antioxidant activity was assessed both directly on YAW samples and on the end products of in vitro digestion using four different cell-free methods. Furthermore, the effect of the smallest fraction (< 3 kDa) of the digestion products on the cellular antioxidant activity of HT29 cells and on the expression of genes implicated in the antioxidant response in THP-1 macrophage cells was evaluated.

## 2. Materials and Methods

### 2.1. Chemicals and Reagents

All the chemicals and reagents were purchased from Sigma-Aldrich (Saint Louis, MO, USA), unless otherwise stated. Millex-GP 33 mm PES 0.22 μm and Amicon Ultra-4 Centrifugal Filter Devices (3 kDa) were purchased from Millipore (Burlington, MA, USA). 3-(4,5-dimethylthiazol2-yl)-2,5-diphenyltetrazolium bromide (MTT) was purchased from Cayman (Michigan, MI, USA). Trypsin was purchased from PAN-Biotech GmbH (Aidenbach, Germany). Fetal bovine serum (FBS) was purchased from Gibco ThermoFisher Scientific (Waltham, MA, USA). PrimeScript RT Reagent Kit (Perfect Real Time) was purchased from Takara Bio (Shiga, Japan). DNase I (RNase-Free) was purchased from New England Biolabs (Ipswich, MA, USA). NucleoZOL was purchased from Macherey-Nagel (Düren, Germany). FastGene IC Green 2× IC Green qPCR Universal Mix was purchased from Nippon Genetics (Tokyo, Japan).

### 2.2. Collection and Preparation of Samples

After a thorough search for YAW samples from dairy companies in Greece, 33 YAW samples were obtained, of which 20 were derived from bovine milk, 7 from ovine milk and 6 from caprine milk. Concerning the month of yogurt production, YAW samples were divided into two groups: 20 YAWs were obtained between November and April (winter group) and 13 YAWs between May and October (summer group). Crude protein of all 33 samples was determined by the Kjeldahl method (Kjeldahl nitrogen × 6.28) in duplicate [34]. Protein content ranged from 0.09 to 1.2% *w*/*v* and pH ranged from 3.7 to 4.7.

### 2.3. In Vitro Digestion Protocol and Digests’ Fractionation

YAW samples were concentrated 5 times by freeze-drying and appropriate rehydration. Freeze-drying was performed under the temperature conditions of −20 °C to 15 °C with a rate of temperature increase of 5 °C every 4 h, and the maximum difference from shelf to sample was 10 °C. Vacuum pressure was 1 mbar for the duration of the procedure. They were afterwards subjected to the INFOGEST 2.0 method [35] with slight modifications. Briefly, all digestions were performed on the basis of equal protein amounts between the tested samples in order to obtain 0.11% *w*/*v* in the final digests [31]. Next, for the oral phase, 4 mL of pre-warmed simulated salivary fluid was added to 5 mL of each sample. Then, 25 μL of CaCl_2_ (300 mM) and appropriate amounts dH_2_O and NaOH (up to 1 mL) were added to adjust the pH to 7, followed by an incubation for 120 s at 37 °C (without salivary α-amylase) while mixing. For the gastric phase, the resulting solution was diluted with 8 mL of simulated gastric fluid at 37 °C, 1 mL of porcine pepsin solution at final activity of 2000 U/mL and 5 μL of CaCl_2_ (300 mM), and was then filled up to 20 mL with dH_2_O and HCl to reach a pH of 3. Incubation for 120 min at 37 °C under rotation followed. For the intestinal phase, 8 mL of pre-warmed simulated intestinal fluid, 2.87 mL of bile extract at a final concentration of 5 mM, 5 mL of pancreatin such that the final activity of trypsin in pancreatin would be 100 U/mL, and 40 μL of CaCl_2_ (300 mM) were added to the 20 mL of the gastric chyme and filled up to 40 mL with dH_2_O and NaOH. The pH was adjusted to 7 followed by incubation for 120 min at 37 °C under rotation in order to simulate the physiological intestinal digestion environment.

After the completion of the intestinal phase, YAW digests (YAW-Ds) were immediately heated at 85 °C for 10 min and placed on ice to deactivate enzymatic activities. Then, samples were centrifuged at 1200× *g* for 5 min and the supernatants were passed through 0.22 μm sterile polyvinylidene fluoride (PVDF) syringe filters. In order to obtain the fraction between 0 and 3 kDa (YAW-D-P3), membrane filters (Ultracel^®^ low binding regenerated cellulose) with a MWCO of 3 kDa were used. YAW-Ds and YAW-D-P3 were kept at −20 °C until subsequent analyses. In parallel, six replicates of blank digests were also prepared using water instead of food and following the same process of the in vitro digestion protocol. The resultant fractions after digestion are hereby referred as BL-D for blank digest (digestion without YAW) and BL-D-P3 corresponding to the YAW-D-P3 fraction.

### 2.4. Biochemical Assays

#### 2.4.1. Oxygen Radical Antioxidant Capacity (ORAC)

The ORAC assay for the YAW samples, before and after digestion, was performed according to the method of Zulueta et al. [36]. Briefly, a 1:20 dilution was conducted for all samples tested with PBS (75 mM, pH 7.4) to avoid interferences and to be transparent. In each well, 20 μL of each sample, blank (PBS) or standard (Trolox) with 120 μL FL (117 nM in 75 mM PBS, pH 7.4) were added. Then, samples were incubated for 15 min at 37 °C while shaking, followed by the addition of 60 μL AAPH (40 mM) to each well. The fluorescence was measured directly, after the addition of the oxidative reagent AAPH, every 120 s for 40 measurements at 485 nm excitation and 535 nm emission. The automated ORAC method was performed on a VICTOR 2030 counter (Perkin Elmer, Waltham, MA, USA). Known quantities of Trolox (3.125–50 μM) were used to construct a standard curve. Measurement units derived from the assay were μmol Trolox equivalents (TEs)/g protein. Each sample was measured in three technical replicates and the experiment was performed three independent times.

#### 2.4.2. 2,2′-Azinobis (3-ethylbenzothiazoline-6-sulfonic acid) Radical Scavenging Assay (ABTS)

The ABTS radical scavenging activity of the YAW samples, before and after digestion, was detected as reported by Ozgen et al. [37]. Briefly, 2.45 mM sodium persulfate and 7 mM ABTS solution were mixed at a ratio of 1:1 to prepare the ABTS^•+^ solution. Then, incubation for 12–16 h at 25 °C in the dark followed to reach a steady state. After that, the ABTS^•+^ solution was diluted with a sodium acetate buffer (20 mM, pH 4.5) until the absorbance value at 734 nm equaled 0.7. Then, 20 μL of the sample with 230 μL of the ABTS^•+^ were incubated for one hour at 25 °C [38]. Known quantities of Trolox (3.75–100 μM) were used to create a standard curve. The absorbance was read at 734 nm with a Tecan Inifinite M200 Pro plate reader (Männedorf, Switzerland). Measurement units derived from the assay were μmol TEs/g protein. Each sample was measured in three technical replicates and the experiment was performed three independent times.

#### 2.4.3. Ferric Reducing Antioxidant Power (FRAP)

The reducing activity of YAW, before and after in vitro digestion, was measured as reported by Benzie et al. [39] with required adaptations using 96-well microplates. Firstly, a mix of 300 mM sodium acetate with glacial acetic acid (pH 3.6), 20 mM FeCl_3_ and 10 mM TPTZ (in 40 mM HCl) at a ratio of 10:1:1 was carried out to form the ferric-tripyridyltriazine (FeIII-TPTZ) solution and the incubation of the solution at 37 °C for 60 min was followed. Then, 280 μL of the FeIII-TPTZ solution was mixed with 20 μL of the sample, and the absorbance was measured at 590 nm with an Epoch 2 spectrophotometer (Biotek, Winooski, VT, USA). A curve of Trolox was created at a concentration range 0.18–5.88 μM. Measurement units derived from the assay were μmol TEs/g protein. Each sample was measured in three technical replicates and the experiment was performed three independent times.

#### 2.4.4. Potassium Ferricyanide Reducing Power (P-FRAP)

The reducing power assay was performed as reported by Liang et al. [40] with slight modifications. Firstly, YAW samples were diluted 1:4 with water, while YAW-Ds and YAW-D-P3 were diluted 1:2, so that the final absorbance of 700 nm fell within the range of the standard curve of BHT (0–100 μM). Briefly, the sample solution was mixed with fresh K_3_Fe(CN)_6_ (1% *w*/*v*) solution and 0.2 M PBS (pH 6.6) at a ratio 1:1:1. Incubation for 20 min at 50 °C while mixing followed. After the addition of 50 μL of TCA (10% *w*/*v*) and 10 μL of FeCl_3_ (0.1% *w*/*v*), the incubation was resumed for an additional 10 min at 50 °C while mixing. The absorbance was read at 700 nm. Measurement units derived from the assay were μmol BHT equivalents/g protein. Each sample was measured in three technical replicates and the experiment was performed three independent times.

### 2.5. Cellular Assays

#### 2.5.1. Cell Culture and Cell Viability of HT29

HT29 cells, cells of the human colon adenocarcinoma, were cultured in DMEM supplemented with 10% (*v*/*v*) FBS, 100 U/mL penicillin, 100 μg/mL streptomycin, 10 U/mL L-glutamine, 100 μM non-essential amino acids and 1 mM sodium pyruvate in a humified incubator at 37 °C in a 5% CO_2_ atmosphere and passaged with trypsin before reaching confluency. To estimate the effect of H_2_O_2_ on cell survival, cells were seeded at a concentration of 5 × 10^4^ cells/well in a 96-well plate for 24 h and next different concentrations of H_2_O_2_ (0–2 mM) were added in the medium. The subsequent day, the cells were washed twice with PBS. After the additional incubation, approximately 2–3 h, at 37 °C in the presence of MTT (0.5 mg/mL), 100 μL/well DMSO was added. The absorbance was measured at a 570 nm wavelength (Tecan Infinite M200 Pro and the results were expressed as percentage of the absorbance displayed by untreated cells (without H_2_O_2_).

#### 2.5.2. Cellular Antioxidant Activity (CAA) Assay

Intracellular ROS was determined in the HT29 epithelial cell line as reported by Piccolomini et al. [41] with a few modifications. Cells were seeded at 5 × 10^4^ cells/well in 96-well plates for 24 h. Cells were washed and then treated with 0.11% *w*/*v* YAW-D-P3 (concentration refers to YAW-D protein), in the presence or absence of 0.5 mM H_2_O_2_ for 1 day. Subsequently, the cells were washed and treated with 100 μL of DCFH-DA (10 μM in PBS containing 0.2% methanol) for half an hour. Measurements were recorded at 37 °C every 2 min for a total of 40 measurements, with excitation of 485 nm and emission of 535 nm using the VICTOR 2030 multilabel counter. The results were expressed as a % of ROS generation to the untreated cells (in the absence of H_2_O_2_). CAA was measured in triplicate and the experiment was performed three independent times.

#### 2.5.3. Cell Culture, Differentiation and Activation of THP-1

THP-1 cells, cells of a human monocytic leukemia cell line, were maintained in RPMI 1640 supplemented with 10% (*v*/*v*) FBS, 10 U/mL L-glutamine, 1 mM of sodium pyruvate, 100 U/mL penicillin, 100 μg/mL streptomycin and 100 μM of non-essential amino acids at 37 °C with 5% CO_2_. The medium was renewed every 2–3 days depending on the confluency of the cells, and the cells were split at a cell density of 10^5^–10^6^ cells/mL. To induce differentiation into the macrophage-like phenotype, monocytes at a density of 0.8× 10^6^ cells/mL in 12-well plates were incubated with PMA (100 ng/mL) for 48 h [42,43]. Afterwards, the PMA-supplemented medium was aspirated, and cells were washed and then treated for 1 day in PMA-free medium (referred as resting phase). The following day, differentiated macrophages were activated with lipopolysaccharide (LPS; 100 ng/mL) in the presence of 0.011% *w*/*v* YAW-D-P3 (concentration refers to YAW-D protein) or BL-D-P3 for 24 h. Cell treatments were performed in triplicate.

#### 2.5.4. Quantification of Gene Expression

THP-1 cells were treated with YAW-D-P3 as described in Section 2.5.3. Next, attached cells were lysed with NucleoZOL as reported by the manufacturer’s protocol. Genomic DNA was removed using DNase I and pure RNA was recovered by ethanol precipitation. The quantity and purity of RNA was evaluated using a spectrophotometer (Q5000, Quawell Technology Inc., San Jose, CA, USA). First, strand cDNA synthesis was performed with the PrimeScript RT reagent kit (Takara) following the manufacturer protocol. The qPCR protocol and analysis was performed as described by Dalaka et al. [44]. Each reaction was performed in duplicate. The relative gene expression normalized to housekeeping gens was calculated using the model described by Hellemans et al. [45].

### 2.6. Statistical Analysis

All presented data are means ± standard error of means (SEMs) of at least two biological replicates. Normality was tested with the Kolmogorov–Smirnov test and when necessary, data were transformed in logarithmic or normalized form [46] to achieve normal distribution. Afterwards, one-way ANOVA was performed followed by Duncan’s post hoc test. Differences between means were considered significant at *p* < 0.05. All statistical analyses were performed with the SPSS for Windows statistical package program, version 22.0.0. Data were visualized with the GraphPad Prism 8 program.

## 3. Results

### 3.1. Antioxidant Activity of YAW before and after In Vitro Digestion Using Biochemical Assays

According to the data presented in Table 1, the values of antioxidant activity of YAW after in vitro digestion (YAW-D, YAW-D-P3) were greatly augmented as assessed by all the biochemical assays used. The antioxidant activity of YAW samples differed significantly as follows: YAW < YAW-D-P3 < YAW-D (*p* < 0.05), regardless of the assay used. The beneficial effect on the antioxidant capacity of YAW resulting from the digestion process was around 2.5–4.5-fold for the ABTS, FRAP and P-FRAP methods, while an even greater increase (13-fold) was observed for the ORAC-FL method (Table 1). These results suggest that in vitro digestion promoted the release of peptides with greater antioxidant activity.

In line with our results, there are several studies that report greater ORAC values compared to the aforementioned biochemical assays [44,47]. The ABTS assay exclusively measures the ability of antioxidants to act as electron donors to neutralize preformed radicals, while ORAC measures both the antioxidant capacity to inhibit the radical initiation as well as the neutralization of the formed radicals [48]. Also, Clausen et al. [49] concluded that milk proteins had higher values in the ORAC method when compared to the ABTS method due to the respectively higher potencies of their amino acids. However, it should be noted that the observations of Clausen et al. were made for intact whey proteins and not fermented ones. These facts could explain the differences among biochemical methodologies.

Regarding the significantly augmented antioxidant activity after digestion, there are several studies reporting such an effect [50]. Studies that report this effect in milk-derived proteins, namely whey protein isolate or concentrate (WPI and WPC, respectively) after digestion, regardless of the assay used, also exist [51]. Furthermore, a previous review summarizes the ongoing effort on the valorization of dairy by-products as well as their proteins and peptides based on their antioxidant potential, among others [52]. Some important findings by Khan et al. [53] report that both whey proteins and caseins possess antioxidant activity. The antioxidant capacity of YAW, a by-product of Greek strained yogurt, is in the same range as other plant or animal by-products such as hempseed [54] and tuna head [55], respectively.

The observed increase in antioxidant activity of YAW samples after digestion could be attributed to the bioactive peptides released, which subsequently exert their antioxidant roles [56]. It should be noted that since YAW is obtained after a fermentation process, this could result in the increased release of bioactive peptides with antioxidant properties not generated by milk or whey protein digestion [57,58].

The peptides released during the digestion process depend mainly on their specific structural properties, such as amino acid composition, sequence, chain length and hydrophobicity [59,60]. In this regard, peptides of relatively low molecular weight have a better possibility to reach the bloodstream and target organs [61,62]. Such fragments can also be produced by endo- and exopeptidases such as alcalase and flavourzyme, where the selection of the suitable enzyme is crucial [63].

The YAW-D-P3 fraction can explain a large percentage (62 to 88%; Table 1) of the antioxidant capacity of the YAW-Ds in all four biochemical assays used. This considerable antioxidant activity of lower-molecular-weight peptides is in agreement with other works. Low-molecular-weight peptides are positively correlated with the antioxidative properties of different food protein hydrolysates [64,65]. Peng et al. [66] reported that the peptides in the range of 0.1–2.8 kDa of WPI hydrolyzed by alcalase showed the highest in vitro radical scavenging activity. In another study [67], the highest antioxidant ORAC values resulted from the WPC hydrolysate fraction with low-molecular-weight peptides (< 1 kDa). Shazly et al. [68] reported that the fraction < 1 kDa obtained from alcalase-hydrolyzed casein showed higher antioxidant activity when compared to other obtained fractions. Virtanen et al. [69] showed that milk fermentates with a high proportion of peptides from 4 to 20 kDa displayed the highest antioxidant activity as measured by the ABTS assay. Conway et al. [70] reported that buttermilk concentrate gave the highest ORAC values after digestion (1320 μmol TE/g protein), followed by skim milk and whey concentrate (811 and 783 μmol TE/g protein, respectively). Peptides from whey proteins, specifically the major ones, α-La and β-Lg, were mainly responsible for their antioxidant activity.

#### 3.1.1. Effect of Milk Animal Origin

**Figure 1:**
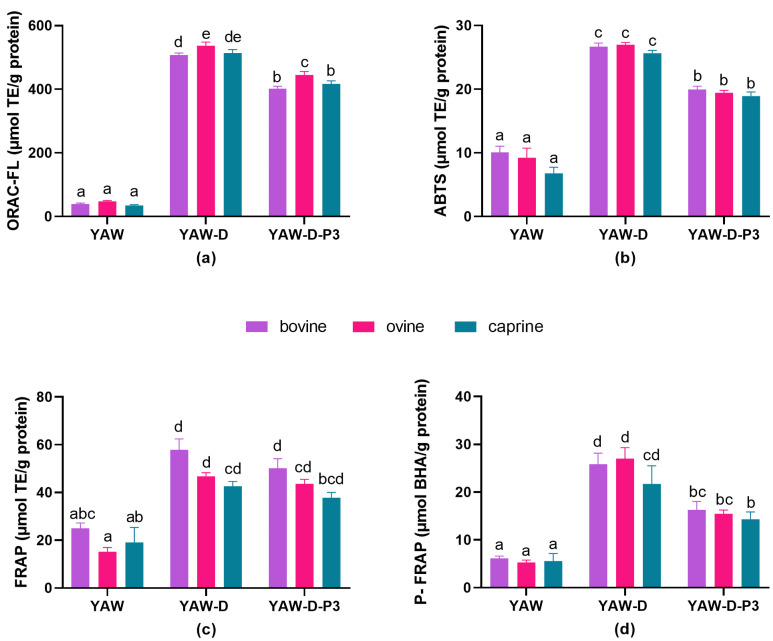
Antioxidant activity of yogurt acid whey from bovine, ovine and caprine milk before (YAW) and after digestion (digested YAW (YAW-D) and digested fraction below 3 kDa (YAW-D-P3) determined by (**a**) oxygen radical absorbance capacity assay (ORAC-FL), (**b**) 2,2′-Azinobis (3-ethylbenzo-thiazoline-6-sulfonic acid) (ABTS), (**c**) ferric reducing antioxidant power assay (FRAP) and (**d**) potassium ferricyanide reducing power (P-FRAP). Results represent mean values of three independent experiments ± SEM. Columns with different letters for each assay differ significantly (*p* < 0.05).

The antioxidant activities of bovine, ovine and caprine YAWs, YAW-Ds and YAW-D-P3s as assessed by biochemical assays are displayed in Figure 1. The ORAC values of YAW were approximately 40 μmol TEs/g protein, without any significant differences among species (*p* > 0.05; Figure 1a). However, the ORAC values of ovine YAW-D (536.8 μmol TEs/g protein) and YAW-D-P3 (445.0 μmol TEs/g protein) were higher compared to their bovine counterparts (*p* < 0.05). The antioxidant capacity of ovine YAW-D-P3 was also higher than that of caprine samples (*p* < 0.05). No statistically significant differences (*p* > 0.05) were found in the ABTS, FRAP or P-FRAP values within the YAW, YAW-D or YAW-D-P3 samples derived from different species (Figure 1b, c and d, respectively).

The effects of species on the antioxidant capacity of milk, its fractions and dairy products have been the subject of investigation of several research groups. However, their results do not support the superiority of any certain species. Simos et al. [71] reported that Prisca goat’s milk, an autochthonous Greek breed, had the highest antioxidant capacity compared to bovine and donkey milk. Revilla et al. [72] analyzed cheese samples made from bovine, ovine or caprine milk by ABTS without detecting any effect of species. However, there is a lack of information about the comparisons after in vitro digestion. Akan et al. [38] evaluated the antioxidant activity of casein and whey fractions of digested camel and donkey milk. They found that the camel casein fraction had the higher activity using the ABTS and CUPRAC methods, while the digested donkey casein fraction had the highest radical scavenging activity using the DPPH method. Tagliazucchi et al. [73] investigated the antioxidant activity of milk of different origins during the in vitro digestion process. Ovine milk revealed stronger ABTS radical scavenging activity compared to bovine, caprine and camel milk after both gastric and intestinal digestion. However, the antioxidant activity of D-P3 did not differ between ovine, caprine and bovine milk, while camel D-P3 showed the lowest ABTS radical scavenging activity. It should be noted though, that this observation was made for D-P3 after normalization to peptide content in the fractions. The species effect reported in the abovementioned studies is not consistent among studies. This could be partially attributed to the different ratios of the milk proteins and the slight differences in the sequences between species. The latter could be the reason for the existence of different peptides (as well as bioactive peptides) after digestion between the species. Although the results of the abovementioned studies could help the interpretation of our data, it is obvious that YAW and its digestion products may have different peptides than milk and other dairy products’ due to the extra fermentation step.

#### 3.1.2. Effect of Seasonality

**Figure 2:**
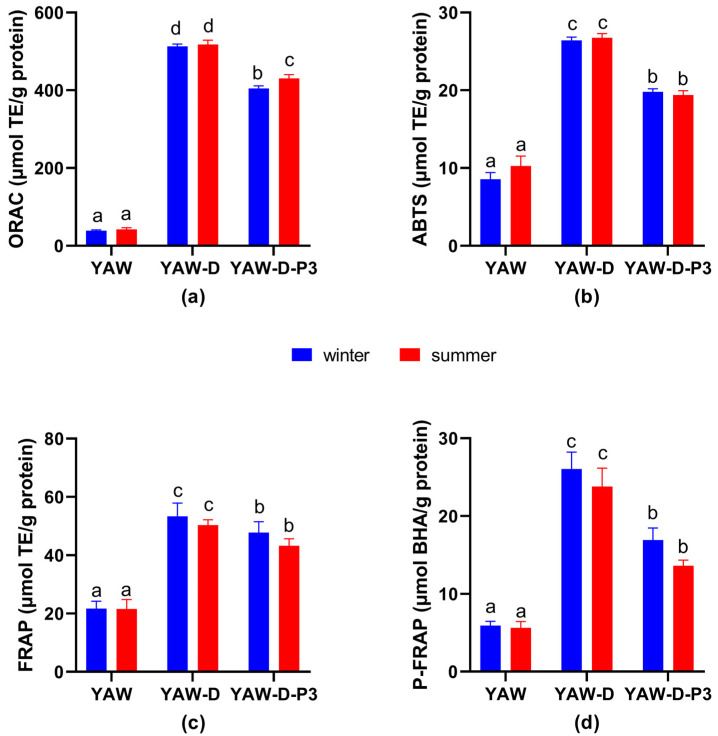
Antioxidant activity of yogurt acid whey collected in winter and summer months before (YAW) and after digestion (digested YAW (YAW-D) and digested fraction below 3 kDa (YAW-D-P3) determined by (**a**) oxygen radical absorbance capacity assay (ORAC-FL), (**b**) 2,2′-Azinobis (3-ethylbenzo-thiazoline-6-sulfonic acid) (ABTS), (**c**) ferric reducing antioxidant power assay (FRAP) and (**d**) potassium ferricyanide reducing power (P-FRAP). Results represent mean values of three independent experiments ± SEM. Columns with different letters for each assay differ significantly (*p* < 0.05).

Figure 2 shows the effect of the season on the antioxidant activities of digested and non-digested YAW samples. ORAC values for YAW-D-P3 were higher (431 μmol TEs/g protein) for samples collected in summer months compared to the winter group (*p* < 0.05; Figure 2a). However, no statistically significant differences (*p* > 0.05) were observed in the three ET methods, ABTS, FRAP and P-FRAP (Figure 2b–d). Although studies dealing with the effect of season on the antioxidant activity of YAW do not exist, our results are in line with what is reported for milk and cheese. Chávez-Servín et al. [74] reported an augmentation of the antioxidant capacity, as assessed by FRAP, of caprine milk and milk whey during the dry season (corresponding to the summer months in our study) compared to those of the rainy season. Santillo et al. [75] reported that cheese digests (corresponding to our YAW-Ds) showed higher antioxidant activity for cheeses made from milk collected in summer months compared with spring months as measured by the DPPH assay. Revilla et al. [72] also reported a significant effect (*p* < 0.05) of the period of raw milk collection on the antioxidant capacity (ABTS assay) of cheese, with higher values reported for summer cheeses.

### 3.2. Cellular Antioxidant Activity of YAW-D-P3

In order to define the safest concentration of H_2_O_2_ for the CAA assay, the cell viability of HT29 cells was assessed by the MTT assay in a range of H_2_O_2_ concentrations between 0–2 mM. The 0.5 mM concentration resulted in about 87% viability, while 1 mM resulted in less than 80% viability. Taking into consideration that in the literature, the H_2_O_2_ concentrations used are commonly between 0.25–0.7 mM [41,76], we decided to perform all subsequent experiments using 0.5 mM H_2_O_2_.

Given that digests can be cytotoxic for HT29 cells [77], to determine a safe amount of provided YAW-D-P3, the HT29 cells were incubated for 1 day to a range of concentrations of YAW-D-P3 (0.0055–0.11% *w*/*v*; concentration refers to YAW-D protein) and their cytotoxic effects were assessed by MTT. The results showed that none of the concentrations of YAW-D-P3 was cytotoxic (Supplementary Data, Figure S1). Thus, the maximum concentration was used in the subsequent experiments for assessing CAA, as it did not affect the viability of HT29 cells. Several studies report a dose–response increase in CAA by exposure to whey proteins in different cell lines as summarized in a review by Corrochano et al. [15]. Kleekayai et al. [78] reported the ROS generation in AAPH-stressed HepG2 cells treated with 1 to 10 mg/mL whey protein hydrolysates to be in the extended range of 20% to 78%, dependent not only on the concentration of whey protein hydrolysates but also on the enzymes used for hydrolysis (Debitrase and FlavorPro Whey) and pH conditions (pH-stat and non-pH-controlled) during the process.

Only peptides resistant to the digestive process are considered bioaccessible, being able to act on intestinal cells promoting antioxidant effects at the cellular level and to show an ameliorative potential against H_2_O_2_-induced oxidative stress [79]. According to the literature, low-molecular-weight peptides have been associated with an increased antioxidant activity [66,80,81]. Furthermore, our results from the biochemical assays showed that the YAW-D-P3 fraction could explain a considerable part of the antioxidant activity of the digested YAW. For the above reasons, only the YAW-D-P3 fraction was selected for the estimation of ROS % inhibition in HT29 cells.

**Figure 3:**
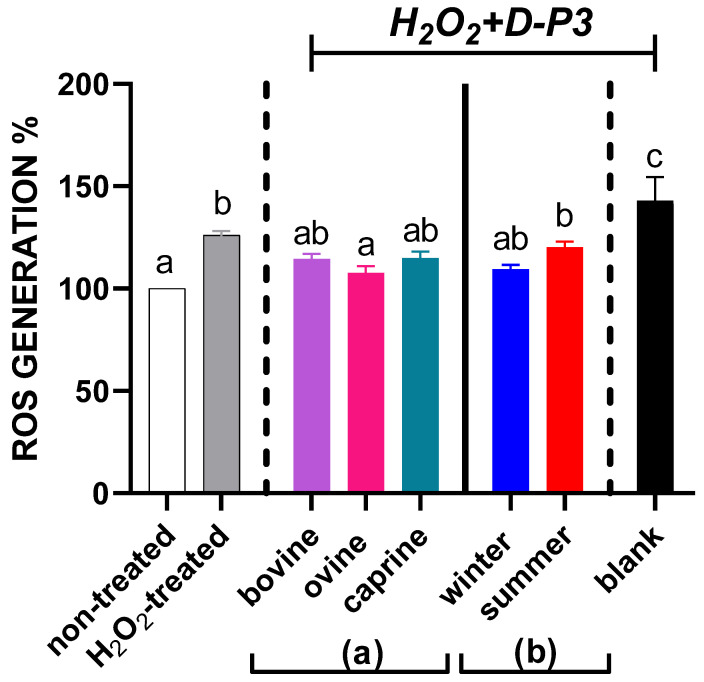
Effect of (**a**) milk animal origin and (**b**) seasonality on reactive oxygen species (ROS) % generation in HT29 cells (seeded at 5 × 10^4^ cells/well) after 1-day exposure to yogurt acid whey (0.11% *w*/*v* refers to starting YAW-D protein) with digested fraction consisting of peptides below 3 kDa (YAW-D-P3). Non-treated cells (cells without H_2_O_2_), H_2_O_2_-treated (cells treated with 0.5 mM H_2_O_2_) and blank (BL-D-P3 treated with 0.5 mM H_2_O_2_) are common for both species-associated and seasonal parts of the diagram. Cell experiments were performed in triplicate at three independent times. Values are presented as means ± SEM. Columns with different letters differ significantly (*p* < 0.05).

Figure 3 shows that cell treatment with the digestion products of YAW significantly decreased radical formation in H_2_O_2_-treated cells compared to the blank digests (*p* < 0.05). Concerning the species effect, only the ovine YAW-D-P3 significantly reduced the radical formation (by 18.3%) compared to the H_2_O_2_-treated (*p* < 0.05). The ROS levels for the cells incubated with YAW-D-P3 collected from winter and summer months did not differ from those treated with H_2_O_2_ (*p* > 0.05).

Foods other than non-dairy products have already been studied using the CAA assay in an overall setting (concerning in vitro digestion and oxidative agent) similar to ours and have been reported to exert significant antioxidant effects both as digests and fractionated digests, which is in line with our data [82,83]. According to current knowledge, there are only a few studies evaluating CAA in epithelial cell lines for milk proteins and dairy products after in vitro digestion. Espindola et al. [24] reported that digested whey protein (0.01–1 mg/mL) in H_2_O_2_-treated Caco-2 cells prevented ROS generation in a dose-related manner, further supporting our observation of a positive effect of the YAW digests. Corrochano et al. [84] evaluated the CAA of WPI, b-LG and a-LA, after simulated gastrointestinal digestion using HT29 as well as Caco-2. The digested b-LG and a-LA, which also exist in YAW [5], efficiently inhibited ROS formation in HT29 cells, but not in Caco-2 cells. However, it is noteworthy that they did not observe such an effect for WPI digests in any of the cell lines. On the contrary, Ma et al. [85] reported an improved CAA of digested WPI compared with both the blank and H_2_O_2_ group in Caco-2 cells, which is in line with our results. Finally, our group has reported that the 0–3 kDa fraction of in vitro digested sweet whey of ovine origin augmented (*p* < 0.05) the CAA compared to H_2_O_2_ in HT29 cells [44]. Similar to our present results for YAW, the 0–3 kDa fractions of sweet whey of bovine and caprine origin did not exert such an effect.

### 3.3. Effect of YAW-D-P3 on Expression of Antioxidant Genes

**Figure 4:**
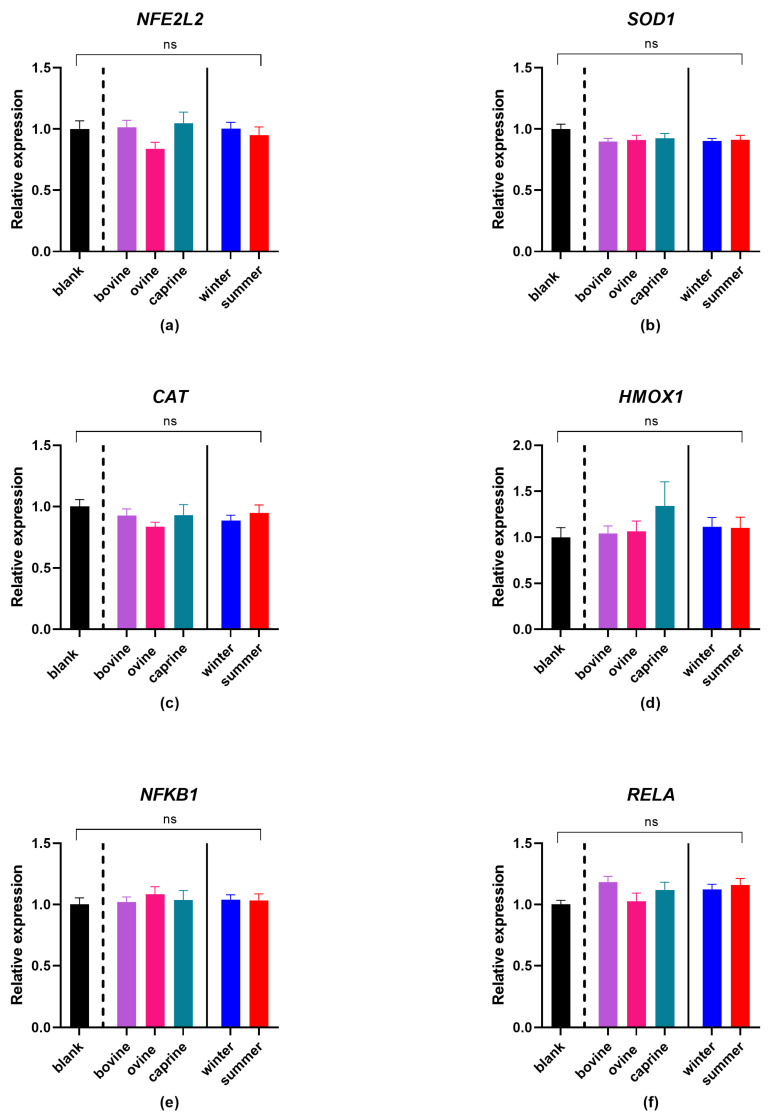
Effect of YAW-D-P3 on LPS-induced mRNA expression in THP-1 cells, compared both with BL-D-P3. THP-1 cells were pretreated with PMA for 48 h (100 ng/mL), 24 h rest, and then were treated with LPS (100 ng/mL) in the presence of YAW-D-P3 (0.011% *w*/*v* refers to starting YAW-D protein) or BL-D-P3 for 24 h. (**a**) *NFE2L2*, (**b**) *SOD1*, (**c**) *CAT*, (**d**) *HMOX1*, (**e**) *NFKB1* and (**f**) *RELA* gene expression levels measured by qPCR. Data are represented as means ± SEM. ns = not significant (*p* > 0.05).

Figure 4 shows the quantification of transcription levels of genes relevant to antioxidant activity. No statistically significant differences were observed between the YAW-D-P3 samples either between different species, seasonality or with BL-D-P3-treated cells regarding the *NFE2L2*, *SOD1*, *NFKB1* and *RELA* expressions (*p* > 0.05).

Contrasting results regarding the effect of milk-whey-related digests on the expression of oxidative-stress-related genes have been reported in previous studies in different cell lines. Xu et al. [86] evaluated the antioxidant effect of whey proteins on H_2_O_2_ toxicity using the myoblast cell line C2C12. In line with our results, *NFE2L2* gene expression was not different (*p* > 0.05) between control and whey proteins. However, an increase in *HMOX-1* gene expression was reported for whey proteins. We previously evaluated the effect of sweet whey on the expression of antioxidant genes [44] and observed that the 0–3 kDa fraction increased the expression of *SOD1*, *CAT*, *NFKB1* and *RELA* depending on the origin of milk. Corrochano et al. [84] reported that the exposure of HT29 cells to digested whey samples did not alter mRNA levels of *SOD1* and *CAT* compared to untreated cells. However, mRNA levels of *SOD1* and *CAT* were greater for blank digest compared to digested whey. It should be noted though, that no oxidative stress factor was used in that study. Kerasioti et al. [87] observed that incubation of the C2C12 muscle cell line with 3.12 mg/mL ovine WPC for 24 h, in the absence of oxidative agents, had no effect on SOD1, CAT and HMOX-1 protein. Conversely, under the same experimental conditions, treatment with ovine WPC led to the augmentation of SOD1 protein levels in the EA.hy926 endothelial cell line.

Although the use of different foods/compounds could be a factor explaining the differences in the reported results between the abovementioned studies, additional factors such as the antioxidant activity of the test compound, the cell line tested, the redox status of the cells before treatment, as well as the incubation time and concentration of oxidative agent and the concentration of the compound used [88], could also explain these differences. Furthermore, the classification of genes and their products as antioxidants or pro-oxidants should not be considered a dichotomy, since this frequently depends on the system conditions, and well-known antioxidants show diverse results under different stimuli [89].

Clinical trials in humans or experimental animal studies including whey products in their diets are of the greatest value in assessing their potential antioxidant properties. Studies reporting the antioxidant activity of whey protein using in vivo models are rather limited. Kim et al. reported that whey protein ameliorated the oxidative changes induced by iron overload, and higher levels of the erythrocyte glutathione were observed in mice fed with whey protein under oxidative stress [90]. Furthermore, Athira et al. [91] reported the beneficial effect of whey protein hydrolysates in oxidative stress mice. A significant increase in liver CAT and SOD levels and a decrease in alkaline phosphatase and creatinine concentration, considered as oxidative biomarkers, was observed.

## 4. Conclusions

To the best of our knowledge, this is the first work thoroughly evaluating the antioxidant capacity before and after in vitro digestion of YAW, using a range of methods. From the obtained results concerning in vitro digestion, it is obvious that the antioxidant values of the YAWs were significantly lower than those of the digested YAWs. Also, the smaller fraction of the digested YAWs could explain a considerable part of the antioxidant activity. The ovine YAWs after in vitro digestion showed an enhanced antioxidant activity compared to their caprine and bovine counterparts by a HAT assay, namely ORAC. Furthermore, only ovine YAW-D-P3 provided protective ability in stress-induced HT29 cells by inhibiting the accumulation of intracellular ROS. The YAW-D-P3 fractions of samples collected between May and October presented a higher antioxidant activity than those collected between November and April as assessed by the ORAC assay, but not with the other three biochemical assays used. However, the above observations could not be coupled to the expression of relevant genes. Although, our results show a rather positive effect of YAW on antioxidant activity, which is consistent with results in the literature on whey and dairy products. Stability assessment experiments and in vivo studies would be essential for safely concluding on the potential use of YAW as an antioxidant in food or feed. Further investigation is also considered necessary for a comprehensive evaluation of the peptides’ sequences from YAW with potential antioxidant activity in vivo that could also elucidate the basis of the observed species effect.

## Figures and Tables

**Table 1:** Antioxidant activity by ORAC-FL (oxygen radical absorbance capacity), ABTS (2,2′-azinobis (3-ethylbenzothiazoline-6-sulfonic acid)), FRAP (ferric reducing antioxidant power) and P-FRAP (potassium ferricyanide reducing power) of yogurt acid whey before (YAW) and after in vitro digestion (YAW-Ds and YAW-D-P3) regardless of milk origin.

Method (Units)	YAW	YAW-D	YAW-D-P3
ORAC-FL (μmol TEs/g protein)	39.9 ± 1.9 ^a^	514.8 ± 5.3 ^c^	418.1 ± 5.2 ^b^
ABTS (μmol TEs/g protein)	9.2 ± 0.7 ^a^	26.5 ± 0.3 ^c^	19.6 ± 0.3 ^b^
FRAP (μmol TEs/g protein)	21.6 ± 2 ^a^	52.3 ± 2.9 ^c^	46.1 ± 2.5 ^b^
P-FRAP (μmol ΒHA eqv/g protein)	5.8 ± 0.4 ^a^	25.2 ± 1.6 ^c^	15.7 ± 1.1 ^b^

Values are means ± SEM (*n* = 33). Mean values in each row with different superscripts differ significantly (*p* < 0.05).

## Data Availability

Data is contained within the article and Supplementary Material.

## Supplementary Materials

The following supporting information can be downloaded at: https://www.mdpi.com/article/10.3390/antiox12122130/s1, Figure S1: Cell viability (MTT assay) after treatment with different concentrations of YAW-D-P3.
