# Supplementary material for: Effect of Milk Origin and Seasonality of Yogurt Acid Whey on Antioxidant Activity before and after In Vitro Gastrointestinal Digestion
Source: Antioxidants (Basel). 2023 Dec 18;12(12):2130. doi: 10.3390/antiox12122130 (PMC10740864; doi:10.3390/antiox12122130)
Supplement: Supplementary file 1 [file antioxidants-12-02130-s001.zip › antioxidants-2711596-supplementary.pdf]

antioxidants

MDPI

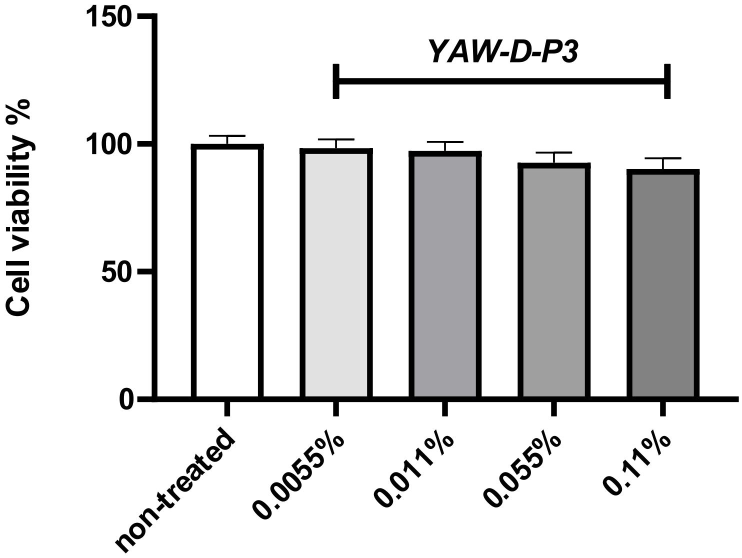

Figure S1. Cell viability (MTT assay) after treatment with different concentrations of YAW-D-P3 (0.0055–0.11% w/v; concentration refers to YAW-D protein). HT29 (5×10^4 cells/well) were grown in 96-well plates for 24 hours. Non-treated cells were set at 100% cell viability and the results of YAW-D-P3 are expressed as the percentage of non-treated cells.
